# Trends and predictors of mortality in unstable pelvic ring fracture: a 10-year experience with a multidisciplinary institutional protocol

**DOI:** 10.1186/s13017-019-0282-x

**Published:** 2019-12-27

**Authors:** Hsien-Te Chen, Yu-Chun Wang, Chen-Chou Hsieh, Li-Ting Su, Shih-Chi Wu, Yuan-Shun Lo, Chien-Chun Chang, Chun-Hao Tsai

**Affiliations:** 1 Department of Orthopedic Surgery, China Medical University Hospital, Taichung, Taiwan; 2 Spine Center, China Medical University Hospital, China Medical University, Taichung, Taiwan; 3 Department of Sports Medicine, College of Health Care, China Medical University, Taichung, Taiwan; 4 Department of Surgery, China Medical University Hospital, Taichung, Taiwan; 5 Department of Surgery, School of Medicine, China Medical University, Taichung, Taiwan; 6 Division of Emergency Disease Surgery, China Medical University Hospital, Taichung, Taiwan; 7 Department of Orthopedic Surgery, School of Medicine, China Medical University, #91 Hsueh-Shih Road, Taichung, 404 Taiwan

**Keywords:** Pelvic fractures, Trauma, Mortality, Risk factors, Institutional protocol, Angioembolization, Pelvic packing

## Abstract

**Background:**

Pelvic ring fracture is often combined with other injuries and such patients are considered at high risk of mortality and complications. There is controversy regarding the gold standard protocol for the initial treatment of pelvic fracture. The aim of this study was to assess which risk factors could affect the outcome and to analyze survival using our multidisciplinary institutional protocol for traumatic pelvic ring fracture.

**Material and methods:**

This retrospective study reviewed patients who sustained an unstable pelvic ring fracture with Injury Severity Score (ISS) ≥ 5. All patients were admitted to the emergency department and registered in the Trauma Registry System of a level I trauma center from January 1, 2008, to December 31, 2017. The annular mortality rate after the application of our institutional protocol was analyzed. Patients with different systems of injury and treatments were compared, and regression analysis was performed to adjust for factors that could affect the rate of mortality and complications.

**Results:**

During the 10-year study period, there were 825 unstable pelvic ring injuries, with a mean ISS higher than that of other non-pelvic trauma cases. The annual mortality rate declined from 7.8 to 2.4% and the mean length of stay was 18.1 days. A multivariable analysis showed that unstable initial vital signs, such as systolic blood pressure < 90 mmHg (odds ratio [OR] 2.53; confidence interval [CI] 1.11–5.73), Glasgow Coma Scale < 9 (OR 3.87; CI 1.57–9.58), 24 > ISS > 15 (OR 4.84; CI 0.85–27.65), pulse rate < 50 (OR 11.54; CI 1.21–109.6), and diabetes mellitus (OR 3.18; CI 1.10–9.21) were associated with higher mortality. No other specific system in the high Abbreviated Injury Scale increased the rates of mortality or complications.

**Conclusion:**

Poor initial vital signs and Glasgow Coma Scale score, higher ISS score, and comorbidity of diabetes mellitus affect the mortality rate of patients with unstable pelvic ring fractures. No single system of injury was found to increase mortality in these patients. The mortality rate was reduced through institutional efforts toward the application of guidelines for the initial management of pelvic fracture.

## Introduction

The pelvic ring, composed of the sacrum and two innominate bones and kept stable by the surrounding sacra-tuberous and sacra-spinous ligamentous structures, protects the neurovascular and hollow visceral structures of the pelvis [1]. Fracture of the pelvic ring is a relatively rare type of fracture, accounting for 1.5–3% of cases and usually related to high energy trauma [2, 3]. It is associated with high mortality and complication rates, and it has been termed “The Killing Fracture” [4]. The major cause of death in patients who sustained a pelvic ring fracture is massive bleeding [5].

Pelvic fractures are often linked to multiple associated injuries. Giannoudis et al. performed a study involving 11,149 patients with traumatic pelvic fracture and found that 21% and 17% of patients had severe chest trauma and head injury, respectively, both of which contributed to mortality [6]. A population-based study in Sweden revealed that traumatic brain injury, advanced age (> 70 years), and Glasgow Coma Scale (GCS) rating < 8 were predisposing factors to higher mortality in patients with pelvic fracture [7].

Currently, there exists some controversy regarding the lethality of pelvic fractures. Trauma patients with pelvic fractures often have multiple injuries, rendering it difficult to distinguish which predictors of mortality are related to the pelvic fracture per se or to certain associated injuries. The first aim of the present study was to identify prognostic factors and evaluate the impact of associated injuries on mortality and complications in patients with pelvic ring fractures.

A multidisciplinary team approach is critical for the management of pelvic trauma to resuscitate the patient, prevent complications, and control bleeding at the time of initial admission to the hospital. An integrated management involving a trauma surgeon, orthopedic surgeon, interventional radiologist, and intensive care unit (ICU) specialist under clinical practice guidelines was developed in the previous decades [8–16]. Black et al. [8] reported decreasing mortality rates over 13 years after initiation of a multidisciplinary institutional protocol. At present, there are no distinct, comprehensive guidelines; rather, these differ between hospital facilities and regional medical systems. In 2008, we initiated an institutional protocol for early decision-making in the treatment of pelvic fracture. The second objective of the present study was to analyze the mortality rate recorded within the previous 10 years while applying this practical guideline for traumatic pelvic ring fracture.

## Materials and methods

### Study design

Registry-based, retrospective, observational cohort study.

### Data collection

The China Medical University Hospital (Taichung, Taiwan) is a 2000-bed facility and a level I trauma center that provides 24 h on duty team of trauma surgeon, orthopedic surgeon, and interventional radiologist to trauma patients; it serves a population of approximately 3 million residents in central Taiwan [17, 18]. Annually, approximately 2500 trauma patients and 600 major trauma patients with an Injury Severity Score (ISS) ≥ 16 are hospitalized through the emergency department (ED).

**Fig. 1:**
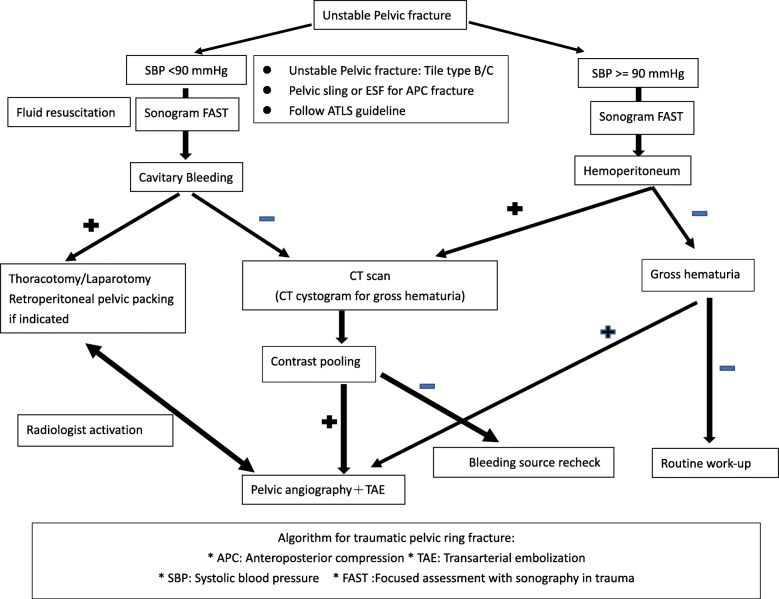
Algorithms for the management of mechanical unstable pelvic fractures based on initial hemodynamic stability

This retrospective study reviewed all hospitalized patients who sustained a pelvic fracture registered in the Trauma Registry System of a level I regional trauma center from January 1, 2008, to December 31, 2017. We included only patients aged > 16 years who sustained trauma, and had partially stable or unstable pelvic fractures as shown through X-ray examination. The inclusion criteria included patients with a trauma International Classification of Diseases, 9th Revision, Clinical Modification code in the range of 800.0–808.9 and a calculated ISS ≥ 5. We applied an institutional protocol involving a multidisciplinary team approach for the timely evaluation and management of patients with pelvic fracture (Fig. 1). All injury data were coded according to the 1998 version of the Abbreviated Injury Scale (AIS). Detailed patient information consisted of the following: age; sex; referral; initial GCS in the ED; vital signs upon arrival at the ED; initial resuscitation method at the ED, including airway intubation, cardiopulmonary resuscitation angiography-embolization (AE), and preperitoneal pelvic packing (PPP); AIS severity score for each body region; ISS; ED disposition (ward, ICU, operation room); hospital length of stay (LOS); LOS in ICU; diabetes mellitus (DM); and in-hospital mortality.

The first aim of this study was to analyze the initial medical parameters associated with mortality rate and complications in the hospital, including respiratory failure, pulmonary edema, adult respiratory distress syndrome, pneumonia, gastrointestinal bleeding, acute renal injury, urinary tract infection, and infection (sepsis, bacteremia, etc.). The parameters for risk assessment included initial vital signs, GCS, initial resuscitation method, ISS score, and sub score. The second aim of the study was to assess the trends in mortality over time among patients with pelvic fractures after application of the clinical practice guideline.

This study was approved by the institutional review board of the China Medical University Hospital. Since the data were analyzed anonymously, informed consent was not required.

### Statistical analysis

Distributions of categorical demographics and comorbidities are presented as raw numbers and percentages (%). We used multiple logistic regression analysis to estimate the odds ratio (OR) and the 95% confidence interval (CI) of mortality and complications associated with patient characteristics. Simple linear regression analysis was for initial treatment, mortality, complications, total hospital LOS (length of stay), and ICU LOS. We used the statistical package SAS version 9.4 (SAS Institute, Cary, NC, USA) to perform all data analyses. A *p* value < 0.05 was considered to denote statistical significance.

## Results

**Fig. 2:**
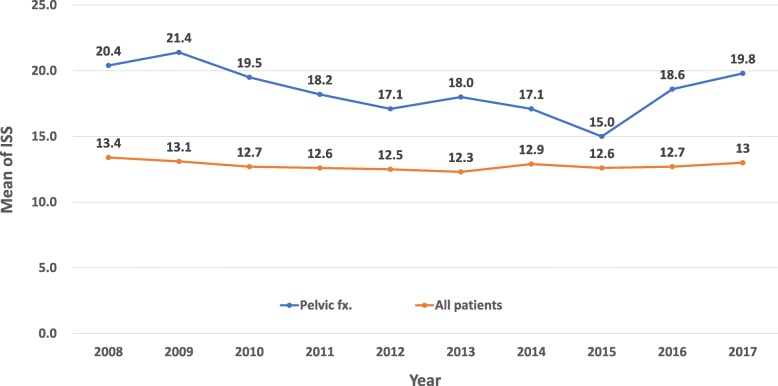
The average ISS score in cases with unstable pelvic fracture was markedly higher than in other trauma cases without pelvic injury during 2008–2017

A total of 825 patients with unstable pelvic ring injuries that met the inclusion criteria from January 1, 2008, to December 31, 2018, were enrolled out of a total database population of 21,371 patients (Table 1). All 825 patients were treated following the algorithm for traumatic pelvic ring fracture (Fig. 1). There are 400 patients of pelvic fracture with ISS ≥ 16 included in our study group. The mean ISS of pelvic fracture patient with ISS ≥ 16 was 27.7. The mean ISS of all patients with ISS ≥ 16 was 21.6. Table 1 shows the characteristics of these patients. The majority were males and middle-aged; 144 (17.5%) patients were initially intubated. The comorbidity of DM accounted for 100 cases (12.1%) and 212 (25.7%) patients received pelvic angioembolization or/and preperitoneal packing within 24 h following admission (Table 2). The mean LOS in the ICU and hospital was 11.9 ± 16 days (mean ± standard deviation [SD]), and 18.1 ± 19.6 days (mean ± SD), respectively. The overall and early mortality rates within 48 h were 5.5% (45 patients) and 3.9% (32 patients), respectively (Table 3). The complication rate was 16.4%, with the most common being respiratory failure, accounting for 9.9% of all patients. The mean ISS score of patients with pelvic ring fracture was 18.4, which was higher (mean value: 12.5) than that of all trauma patients (ISS ≥ 5) during the 10-year period (*p* < 0.01) (Fig. 2).

**Table 1:** Characteristics of patients (aged > 16 years) with pelvic ring fractures (ISS ≥ 5) at CMUH from 2008 to 2017

	Pelvic ring injuries *n* = 825	
Characteristics	*n*	%
Age (years)		
16–25	165	20.0
26–45	243	29.5
46–65	261	31.6
66–75	80	9.7
> 75	76	9.2
Male sex	456	55.3
From Referral	375	45.5
ED GCS score		
> 8	732	88.7
≤ 8	93	11.3
ED GCS motor score		
6	686	83.2
5–2	95	11.5
1	44	5.3
ISS		
5–15	425	51.5
16–24	175	21.2
25–35	158	19.2
> 35	67	8.1
AIS		
Head/neck score > 2	166	20.1
Chest score > 2	231	28.0
Abdomen score > 2	164	19.9
Extremity score > 2	576	69.8
Intubated		
None	681	82.5
In other hospital	41	5.0
In our hospital	103	12.5
ED systolic blood pressure		
> 90 mmHg	723	87.6
61–90 mmHg	87	10.6
≤ 60 mmHg	10	1.2
Missing	5	0.6
ED pulse		
51–120 bpm	733	88.8
> 120 bpm	80	9.7
0–50 bpm	7	0.9
Missing	5	0.6
DM	100	12.1

**Table 2:** In-hospital processing measures

	Pelvic ring injuries	
Process measures	*n*	%
Nonoperative	613	74.3
Angioembolization	182	22.1
Preperitoneal packing	13	1.6
Angioembolization + preperitoneal packing	17	2.1
ICU	137	16.6
Operating room	415	50.3
ICU LOS, mean ± SD	11.9 ± 16.0	
Hospital LOS, mean ± SD	18.1 ± 19.6

**Table 3:** Number of deaths and adverse events among patients with pelvic ring fractures between 2008 and 2017

	Pelvic ring injuries	
Outcomes	*n*	%
Mortality	45	5.5
Early mortality (< 48 h)	32	3.9
Complications	135	16.4
Respiratory failure	82	9.9
Pulmonary edema	5	0.6
ARDS	8	1.0
Pneumonia	27	3.3
GI bleed	12	1.5
Acute renal failure	7	0.9
UTI	31	3.8
Compartment syndrome (extremity)	1	0.1
Osteomyelitis	1	0.1
Other infection (sepsis, bacteremia, etc.)	18	2.2

The patients who required preperitoneal packing were at higher risk of mortality (OR 36.19; 95% CI 11.52–113.67; *p* < 0.0001) than those who needed angioembolization (OR 4.23; 95% CI 2.06–8.68; *p* < 0.0001) (Table 4). The patients who received both preperitoneal packing and angioembolization were at the highest risk of complications (OR 6.96; 95% CI 2.96–16.38; *p* < 0.0001). The risk of mortality included initial vital signs with GCS ≤ 8 (OR 3.87, 95% CI 1.57–9.58, *p* < 0.0001), systolic blood pressure (SBP) ≤ 60 mmHg (OR 9.48; 95% CI 1.85–48.52; *p* < 0.0001), and pulse rate 0–50 bpm (OR 11.54; 95% CI 1.21–109.6; *p* < 0.0001) (Table 5). The risk of complications included initial vital signs, with 60 < SBP < 90 mmHg (OR 2.23.19; 95% CI 1.29–3.86; *p* < 0.0001) and pulse rate > 120 bpm (OR 1.91; 95% CI 1.07–3.44, *p* < 0.0001). Higher ISS score was also related to higher rates of mortality (ISS > 35; OR 47.56; 95% CI 6.83–331.1; *p* < 0.0001) and complications (ISS > 35; OR 14.33; 95% CI 5.13–40.0; *p* < 0.0001). In contrast, individual AIS involvement was not linked to higher rates of mortality or complications. Notably, the comorbidity of DM was related to a higher rate of mortality (OR 3.18; 95% CI 1.10–9.21; *p* < 0.0001). In addition, higher ISS scores were also related to longer ICU stay and hospitalization (Table 6). An AIS head/neck involvement score > 2 indicated longer stay in the ICU.

**Table 4:** Simple logistic regression for initial treatment, mortality, and complications

	Mortality (yes vs. no)			Complication (yes vs. no)		
Variable	OR	95% CI	*p*	OR	95% CI	*p*
Treatment			< 0.0001			< 0.0001
Nonoperative	1.00	Reference		1.00	Reference	
Angioembolization	4.23	2.06–8.68*		2.57	1.70–3.87*	
Preperitoneal packing	36.19	11.52–113.67*		5.06	1.75–14.66*	
Angioembolization + preperitoneal packing	14.60	5.00–42.60*		6.96	2.96–16.38*	

*There is significant difference *p* < 0.0001

**Table 5:** Multiple logistic regression for patient characteristics, mortality, and complications

	Mortality (yes vs. no)		Complication (yes vs. no)	
Variable	OR	95% CI	OR	95% CI
ED GCS				
> 8	1.00	Reference	1.00	Reference
≤ 8	3.87	1.57–9.58*	1.67	0.91–3.08
ED SBP				
> 90 mmHg	1.00	Reference	1.00	Reference
61–90 mmHg	2.53	1.11–5.73*	2.23	1.29–3.86*
≤ 60 mmHg	9.48	1.85–48.52*	0.92	0.21–4.06
ED pulse				
51–120 bpm	1.00	Reference	1.00	Reference
> 120 bpm	0.81	0.32–2.03	1.91	1.07–3.44*
0–50 bpm	11.54	1.21–109.6*	-	-
DM				
No	1.00	Reference	1.00	Reference
Yes	3.18	1.10–9.21*	1.62	0.87–3.03
ISS				
5–15	1.00	Reference	1.00	Reference
16–24	4.84	0.85–27.65*	2.66	1.38–5.14*
25–35	11.97	2.09–68.64*	6.23	2.87–13.5*
> 35	47.56	6.83–331.1*	14.33	5.13–40.0*
AIS				
Head/neck score > 2				
No	1.00	Reference	1.00	Reference
Yes	0.87	0.34–2.26	1.09	0.61–1.96
Chest score > 2				
No	1.00	Reference	1.00	Reference
Yes	0.66	0.28–1.56	0.52	0.30–0.92*
Abdomen score > 2				
No	1.00	Reference	1.00	Reference
Yes	1.63	0.67–3.94	1.07	0.61–1.86
Extremity score > 2				
No	1.00	Reference	1.00	Reference
Yes	1.26	0.46–3.49	1.13	0.66–1.94

*There is significant difference *p* < 0.0001

**Table 6:** Simple linear regression analysis for total hospital LOS (length of stay) and ICU LOS

	Hospital LOS (*n* = 825)			ICU LOS (*n* = 362)		
	Coefficient	SE	*p*	Coefficient	SE	*p*
ISS (vs. 5–15)			< 0.0001			< 0.0001
16–24	9.27	1.59		2.95	2.49	
25–35	16.90	1.65		7.46	2.35	
> 35	24.37	2.33		13.68	2.76	
AIS						
Head/neck score > 2 (vs. ≤ 2)	11.88	1.65	< 0.0001	5.52	1.73	0.0016
Chest score > 2 (vs. ≤ 2)	6.67	1.50	< 0.0001	0.64	1.69	0.7098
Abdomen score > 2 (vs. ≤ 2)	10.54	1.67	< 0.0001	0.99	1.83	0.5898
Extremity score > 2 (vs. ≤ 2)	2.14	1.48	< 0.0001	1.59	1.81	0.3820

**Fig. 3:**
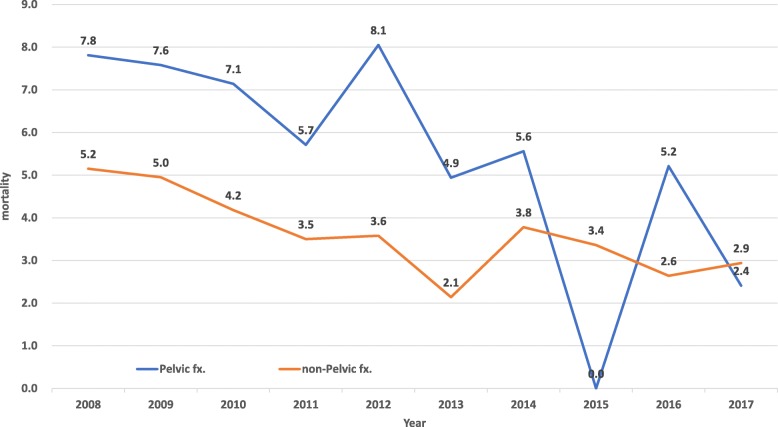
The trends of annual mortality declined from 7.8% to 2.4% over a 10-year period (*p* for trend < 0.05)

Following the application of the institutional clinical protocol for the management of traumatic pelvic ring fracture, the rate of mortality declined annually, from 7.8% in 2008 to 2.4% in 2017 (*p* for trend < 0.05) (Fig. 3). The trends of annual mortality of pelvic fracture with ISS ≥ 16 declined from 14.3 to 2.0% over a 10-year period (*p* for trend = 0.032). The mean mortality of pelvic fracture patient with ISS ≥ 16 was 10.8%. There was no statistically significant difference in the average 10-year mortality rate between patients with unstable pelvic fracture (5.5%) and those with other trauma (whose ISS ≥ 5) without pelvic fracture (3.6%; *p =* 0.0587) (Fig. 3). According to the algorithm for traumatic pelvic ring fracture, there were two indications for emergent stable vital signs, namely contrast extravasation on computed tomography scan (62 patients; 73.8%) and unstable hemodynamics without other cavitary or external bleeding (22 patients; 26.2%). In our hospital, there is an interventional radiology team on-call for 24 h per day. The average time to transarterial embolization (TAE) in our study was 62.0 ± 33.4 min (mean ± SD), with 47 patients (56%) receiving TAE within 1 h. In our study, 22% of cases underwent selective embolization, without occurrence of complications in the entire study population.

## Discussion

In this study, we identified the presence of unstable initial vital signs (i.e., SBP < 90 mmHg, GCS < 9, ISS > 15, pulse rate < 50 bpm) and DM as significant risk factors for mortality or complications in patients with pelvic fracture. We also found an overall OR of 2.0 for the effect of a pelvic fracture on the rate of mortality. The most common type of complication was respiratory complications.

Hemorrhage is considered the leading cause of death in patients with pelvic ring injuries [5, 19, 20]. Lustenberger et al. examined trauma registry reports comparing 3296 patients with pelvic fracture and 59,737 patients without pelvic fracture. Their univariate analysis revealed that the OR of pelvic fracture for mortality was 2.4, which is consistent with our result (OR 2.0) [21]. Some studies suggested brain injuries as the major cause of death in patients with pelvic ring fracture [22–24]. Further investigation demonstrated that isolated pelvic fractures were rarely lethal injuries, while a combination of pelvic and abdominal or thoracic injury resulted in a fatal course [5]. Consequently, we compared the combination injury as assessed using different systems. Our data show that pelvic injuries are linked to a higher mortality rate than other kinds of injuries. In our 10-year analysis, we did not identify any specific association with a single injury type; however, the severity of combined ISS appears to increase the rate of mortality in patients with pelvic trauma. Therefore, in cases with pelvic injuries, injuries at other sites should also be assessed, and a multidisciplinary approach should be taken in the initial assessment and in preventing secondary injuries. The comorbidity of DM contributed to a higher mortality rate. DM may increase the rates of mortality and complications in cases with pelvic trauma through multiple mechanisms. Patients with diabetes who sustain trauma experience higher rates of complications and utilization of resources [11, 12].

During the analyses period of 10 years, the rate of mortality and mean LOS in patients with complex pelvic fracture in our institution was 5.5% and 18.1 days, respectively. These data are consistent with those of another population-based study conducted in Taiwan. Yang et al. reported a 12-year (2000–2011) Taiwanese nationwide health insurance database study [25]. The mean case-fatality rate was 2.1% and 1.6% in male and female patients, respectively; the mean LOS for complex pelvic fracture was 17.9 days. As a level I trauma center in Taiwan, 45.5% of our cases were transferred from other hospitals. The mean ISS score in our ED cases was higher than that reported in other hospitals in Taiwan, which may explain the higher mortality rates [5, 26]. The annual mortality rate declined from 7.8% in 2008 to 2.4% in 2017 (*p* < 0.001). This rate is in line with data obtained from other contemporary studies [8, 26] and lower than that reported in a previous study (overall mortality 7.7%) performed in another level I trauma center [27].

With recent improvements in pre-hospital management and standardized treatment protocols for the treatment of shock, several studies have shown a decreasing mortality rate among trauma patients with pelvic fractures [8–16]. Previous studies have investigated initial treatments for the management of hemorrhage, including temporary pelvic fracture stabilization, AE, PPP and Resuscitative Endovascular Balloon Occlusion of the Aorta (REBOA) [28–36].

Pelvic bleeding is the result of disruption of the presacral venous plexus and bone. Our protocol emphasizes the early implementation of the Advanced Trauma Life Support guideline, as well as the usage of focused assessment with sonography in trauma (FAST), to detect life-threatening signs and ensure timely intervention for hemostasis and resuscitation. Approximately 85% of pelvic fracture hemorrhages are caused by bone and venous bleeding [16, 37]. Therefore, temporary mechanical stabilization methods, such as circumferential sheet wrapping and pelvic packing, are necessary to control bleeding [38–40].

The selection of an external skeletal fixator or a circumferential compression sling/binder device for acute temporary stabilization in pelvic injury remains debatable. In our institution, we use a circumferential compression device/sling as the first choice, as recommended by numerous guidelines (i.e., Advanced Trauma Life Support [41], Eastern Association for the Surgery of Trauma [42], Western Trauma Association [43], and The American College of Surgeons Trauma Quality Improvement Program [44]). Anterior pelvic external fixation is indicated in patients with unstable antero-posterior compression and lateral compression injuries by the Young–Burgess fracture classification [45]. C-clamp is used in cases with stabilized posterior pelvic ring disruption to control hemorrhage, especially in patients with vertical unstable pelvic injury [21]. The Young–Burgess system has been shown to predict mortality [40]; however, there is currently a gap in interobserver reliability in the classification systems of pelvic ring fractures [39]. Moreover, use of the posterior C-clamp is contraindicated in comminuted and transforaminal sacral fractures, fractures of the iliac wing, and lateral compression-type injuries [46]. Therefore, training is required for the selection of the most appropriate external fixation for a particular type fracture. Our system incorporates fewer external pelvic fixations owing to the learning curve of the technique and the need for experience to avoid complications from pin placement.

AE, REBOA, and PPP were effective in controlling hemorrhage [30, 31, 33–36, 47–54]. However, these procedures have been associated with complications such as wound complication in PPP [32, 47, 55], and gluteal muscle necrosis, nerve injury bladder, or ureteral infarction after AE [31, 37, 48, 56, 57], and acute kidney injury, vascular complications in REBOA [58]. Therefore, the optimal management algorithm for the management of hemodynamically unstable patients with pelvic fractures remains controversial. Based on recent literature, the two most common algorithms for the treatment of patients with persistent hemodynamic instability were early AE or early PPP with selective AE [29, 32, 47, 59]. The timing of the procedure is the key for successful intervention and improved survival [35, 36, 59]. The guideline established by the World Society of Emergency Surgery [10] recommends PPP as first-line therapy; however, this recommendation is inconsistent across guidelines [35, 41, 42, 44]. In our institutional guideline, failure of fluid resuscitation and circumferential compression device/sling in the initial stage was followed by application of early TAE. The role of PPP in our protocol was application in case of hemodynamic instability and the unavailability or failure of interventional radiology. In our study, AE effectively controlled hemorrhage, in line with other reports [22, 31, 50, 52]. These results are consistent with those of other investigations; patients who received both TAE and PPP had a higher rate of complications than those receiving another procedure alone [30].

The strength of our study is the long-term detailed database of a level I trauma center, which provided complete data (e.g., initial vital signs, management, length of stay to the ICU, complications, and mortality).

Our results suggest that the pelvic fracture itself should not be considered a fatal fracture as previously described. This statement is also consistent with the conclusion of a post-mortem analysis of 655 patients with pelvic fracture trauma performed by Papadopoulos et al., which suggested that only 3.5% of deaths are directly related to actual pelvic fractures [60]. Our findings indicate that pelvic fracture mortality is subject to a number of confounding factors (especially when part of AIS).

Our study had several limitations. Firstly, there was an inherent selection bias owing to the retrospective design of the study. Secondly, patients without hospital cardiac arrest and declared death at the accident were not included in our hospital trauma registered database, which could lead to bias. Thirdly, this was a single-center database analysis, limited to a single level I trauma center; hence, the findings may not be representative of other populations in other areas. Fourth, REBOA is a bridge to time-consuming procedures. However, as REBOA is not authorized by the Taiwan Food and Drug Administration, we would not be able to compare the effect of REBOA with other methods in our study. Finally, the study design did not include a control group. However, the development of a randomized controlled clinical trial to prove superiority of certain procedures over others in this critical injury setting is challenging.

## Conclusion

The results of our study demonstrated that initial vital signs, ISS, GCS, and DM are associated with a high rate of mortality. Based on our 10 years of experience, the mortality rate was reduced through institutional efforts toward the application of guidelines for the initial management of pelvic fracture.

## Data Availability

Not applicable

## Abbreviations

AIS: Abbreviated injury scale
ED: Emergency department
FAST: Focused assessment with sonography in trauma
GCS: Glasgow Coma Scale
ICU: Intensive care unit
ISS: Injury Severity Score
LOS: Length of stay
PPP: Preperitoneal pelvic packing
REBOA: Resuscitative Endovascular Balloon Occlusion of the Aorta
SBP: Systolic blood pressure
TAE: Transcatheter arterial embolization
